# Distinct Echocardiographic Phenotypes in Primary vs. Secondary Iron Overload Cardiomyopathy: A Pilot Study on Myocardial Work Indices

**DOI:** 10.3390/medsci14020223

**Published:** 2026-04-29

**Authors:** Luis Andrés Vega-Quesada, Zuilma Yurith Vásquez-Ortiz, María Elena Soto-López, Gerardo Marín, Cristofer Zarate-Calderon

**Affiliations:** 1 Department of Echocardiography, Instituto Nacional de Ciencias Médicas y Nutrición Salvador Zubirán, Mexico City 14080, Mexico; luisvegacardiol@gmail.com; 2 Department of Immunology and Rheumatology, Instituto Nacional de Cardiología Ignacio Chávez, Mexico City 14080, Mexico; mesoto50@hotmail.com; 3 Department of Neurosurgery, “Hospital Regional 1° de Octubre”, Instituto de Seguridad y Servicios Sociales de los Trabajadores del Estado (ISSSTE), Mexico City 07300, Mexico; drmarin.neuroscience@gmail.com; 4 Institute of Brain Research, Universidad Veracruzana, Xalapa 91190, Mexico; cristoferjzc@gmail.com

**Keywords:** hemochromatosis, iron overload cardiomyopathy, echocardiography, myocardial work, global work index, global wasted work, global longitudinal strain

## Abstract

**Background:** Iron overload cardiomyopathy (IOC) is a major determinant of outcomes in hemochromatosis, and conventional echocardiography may miss early myocardial toxicity. Comparative data on primary (PH) versus secondary hemochromatosis (SH) using myocardial work (MW) indices are limited. **Methods:** We performed a retrospective cross-sectional study of 34 adults (16 PH and 18 SH patients) at a tertiary center. They all underwent echocardiography with speckle-tracking to obtain LV global longitudinal strain (GLS) and non-invasive MW indices from pressure-strain loops: global work index (GWI), global constructive work (GCW), global wasted work (GWW), and global work efficiency (GWE). Echocardiographic phenotypes were classified as a Normal, Dilated, Restrictive, or right ventricular/pulmonary hypertension (RVPH) phenotype. **Results:** SH patients showed higher iron burden and neurohormonal activation than PH patients (maximum ferritin 2954 vs. 444 ng/mL; BNP 93 vs. 13.5 pg/mL; both *p* < 0.001) and accounted for all deaths (33% vs. 0%) despite similar 3D LVEFs and GLSs. PH patients predominantly exhibited Normal phenotypes (81%), whereas SH patients more often showed advanced phenotypes, mainly RVPH and Dilated. GWI correlated inversely with ferritin (ρ ≈ −0.40), particularly ferritin at echocardiography in SH patients, while PH patients showed no significant correlations. GWW was higher in Dilated/RVPH compared to Normal phenotypes, and in SH patients, higher maximum ferritin was associated with impaired right ventricular free-wall strain. **Conclusions:** PH and SH patients exhibit distinct IOC phenotypes, with SH patients showing more advanced remodeling and worse outcomes. In this exploratory analysis, MW indices showed modest associations with iron burden markers, suggesting they may provide complementary information beyond LVEF and GLS. These preliminary findings require validation in larger, prospective studies.

## 1. Introduction

Hemochromatosis is a complex clinical syndrome characterized by the progressive accumulation of iron in the parenchyma of multiple organs, leading to chronic toxicity and subsequent organ failure [1,2]. As the most common autosomal recessive disorder in Caucasian populations, its global prevalence is estimated at 1 in every 200–300 adults [2]. Iron overload cardiomyopathy (IOC) remains a critical complication; it is the cardiac dysfunction resulting from excessive iron deposition in the myocardium, whether due to hereditary metabolic defects (Primary Hemochromatosis [PH]) or chronic parenteral loading from transfusion-dependent anemias (Secondary Hemochromatosis [SH]) [3,4].

Pathophysiologically, when transferrin saturation is exceeded, non-transferrin-bound iron enters cardiomyocytes, primarily through L-type voltage-dependent calcium channels [4,5]. This intracellular accumulation triggers the production of reactive oxygen species, which impairs excitation-contraction coupling and promotes interstitial fibrosis [5,6]. While early iron toxicity can induce subtle myocardial damage, conventional echocardiographic markers such as Left Ventricular Ejection Fraction (LVEF) often remain within normal ranges until advanced stages of the disease, thereby providing a false sense of clinical stability [6,7]. Furthermore, while historical data suggested that diastolic dysfunction, often presented as a restrictive filling pattern, was an early marker of IOC [8,9], our current understanding recognizes that overt restrictive patterns typically signify established and severe myocardial involvement rather than incipient toxicity [10].

Recent advancements in speckle-tracking echocardiography have enabled the detection of subclinical systolic abnormalities before substantial structural remodeling occurs. Mounting evidence, including pivotal work by Swiatczak et al. [11] and Rozwadowski et al. [10], has demonstrated a consistent inverse relationship between global longitudinal strain (GLS) and systemic iron burden [12]. However, GLS is fundamentally load-dependent, meaning that its values can be confounded by fluctuations in blood pressure and ventricular afterload, which are common in patients with multi-organ involvement [13].

Against this backdrop, non-invasive myocardial work (MW) indices have emerged as a novel diagnostic framework [13]. By integrating brachial blood pressure with strain-derived pressure-strain loops, MW indices offer a more robust characterization of myocardial bioenergetics and metabolic efficiency [13,14]. This approach potentially overcomes the limitations of load-dependent strain, yet its application in differentiating the phenotypic expressions of PH and SH remains largely unexplored.

The aim of this pilot study was to characterize the distinct echocardiographic phenotypes in patients with primary and secondary hemochromatosis and to correlate their cardiovascular profiles with biochemical biomarkers of iron overload. We hypothesized that myocardial work indices, particularly the Global Work Index (GWI), would provide a more sensitive and load-independent marker of subclinical myocardial inefficiency compared to conventional strain, reflecting the cumulative metabolic impact of iron toxicity across different clinical stages.

## 2. Materials and Methods

### 2.1. Study Design and Setting

This retrospective, observational, cross-sectional study was conducted at the Instituto Nacional de Ciencias Médicas y Nutrición Salvador Zubirán (INCMNSZ), a tertiary referral center in Mexico City, Mexico. All patients with a confirmed diagnosis of primary or secondary hemochromatosis who underwent echocardiographic evaluation at INCMNSZ between January 2019 and October 2023 were consecutively screened for eligibility. This study was conducted in accordance with the Declaration of Helsinki, and the protocol was approved by the Institutional Review Board of INCMNSZ (Protocol code: REF.4776, approved on 5 October 2023).

### 2.2. Patient Selection and Definitions

Consecutive patients aged ≥18 years with a confirmed diagnosis of primary (hereditary) or secondary hemochromatosis/iron overload were eligible. Primary hemochromatosis (PH) was diagnosed according to the EASL 2022 Clinical Practice Guidelines on Haemochromatosis [15], requiring elevated transferrin saturation (>45%) and/or hyperferritinemia with confirmatory HFE genotyping (C282Y homozygosity, C282Y/H63D compound heterozygosity, or other pathogenic HFE variants). Secondary hemochromatosis (SH) was defined as iron overload attributable to transfusion-dependent anemias (myelodysplastic syndromes, aplastic anemia, and hemoglobinopathies) or ineffective erythropoiesis, confirmed by the treating hematologist based on clinical history, cumulative transfusion burden, and biochemical iron indices.

Exclusion criteria were congenital heart disease, significant primary valvular heart disease, known coronary artery disease, concurrent infiltrative or autoimmune cardiomyopathy, loss to follow-up without an echocardiographic study from 2019 onward, or incomplete clinical records precluding adequate analysis. The final analytic cohort comprised 34 patients who fulfilled all inclusion criteria. No echocardiographic studies were excluded due to inadequate image quality; all 34 enrolled patients had analyzable data for conventional echocardiographic parameters.

### 2.3. Clinical and Laboratory Data

Demographic variables, relevant comorbidities, etiology of iron overload, and iron-removal strategies (therapeutic phlebotomy and/or iron chelation therapy) were obtained from the clinical record. Laboratory biomarkers were collected when available and included hemoglobin, transferrin saturation, serum ferritin, B-type natriuretic peptide (BNP), and troponin.

Given the temporal variability of ferritin, iron burden was characterized using complementary ferritin definitions derived from available clinical measurements: ferritin at/around the time of echocardiography (“at echocardiography”), maximum recorded ferritin, and the most recent available ferritin value. Ferritin at the time of echocardiography was operationally defined as the serum ferritin value obtained within ±30 days of the echocardiographic examination. When multiple values were available within this window, the measurement closest in time to the echocardiogram was selected.

It should be noted that serum ferritin is an acute-phase reactant influenced by inflammation, hepatic dysfunction, malignancy, and chronic systemic disease [16], and therefore may not reliably reflect myocardial iron content, particularly in the SH subgroup, which includes patients with myelodysplastic syndromes and other chronic hematologic conditions. To partially mitigate the well-known temporal variability and confounding of single ferritin measurements, three complementary ferritin definitions were employed as described above.

Cardiac magnetic resonance (CMR) with T2* mapping was performed in 12 of 34 patients (35%; 6 PH and 6 SH patients) for clinical indications during routine follow-up. However, these studies were not acquired under a standardized research protocol, were performed at variable time points relative to the echocardiographic evaluation and were not uniformly available across the cohort. Including these non-systematic CMR data in the primary analysis would introduce selection bias and temporal misalignment; therefore, CMR T2* values were not incorporated into the present analysis.

### 2.4. Echocardiographic Acquisition

All echocardiographic examinations were acquired using Vivid E9 or Vivid E95 ultrasound systems (GE Healthcare, Horten, Norway) equipped with an M5S phased-array transducer (GE Healthcare, Horten, Norway). Standard two-dimensional and Doppler data were obtained in parasternal long- and short-axis views and apical 4-, 2-, and 3-chamber views. Digital studies were stored for offline analysis using EchoPAC (version 112.5, GE Healthcare, Horten, Norway).

Brachial blood pressure was measured following a standardized institutional protocol based on the 2017 ACC/AHA Guideline for the Prevention, Detection, Evaluation, and Management of High Blood Pressure in Adults [17]. Measurements were obtained with the patient in a seated position after a minimum of 5 min of rest, using a validated automated oscillometric blood pressure monitor (Model HEM-7120, Omron Healthcare, Kyoto, Japan) on the non-dominant arm. The mean of two consecutive readings, obtained at least 1 min apart, was recorded and used for non-invasive myocardial work calculations.

### 2.5. Conventional Echocardiographic Analysis

Cardiac chamber dimensions and volumes were measured in accordance with American Society of Echocardiography/European Association of Cardiovascular Imaging recommendations [18]. LV mass was calculated using the linear method and indexed to body surface area. LVEF was quantified using the biplane Simpson method, and three-dimensional LVEF was obtained for all patients (*n* = 34) using a 4V-D matrix-array transducer (GE Healthcare, Horten, Norway) as 3D acquisition is part of the routine institutional echocardiographic protocol.

Diastolic function was evaluated using transmitral inflow (E and A velocities and E/A ratio) and pulsed-wave tissue Doppler (septal and lateral e′ velocities) to calculate the E/e′ ratio. RV systolic function was assessed using tricuspid annular plane systolic excursion (TAPSE) and RV fractional area change (FAC), when available. Pulmonary artery systolic pressure (PASP) was estimated when tricuspid regurgitation signals allowed.

### 2.6. Myocardial Deformation and Myocardial Work Analysis

Advanced myocardial mechanics were assessed using 2D speckle-tracking echocardiography. All echocardiographic studies were acquired and analyzed by a single experienced echocardiographer (L.A.V.-Q., specialist in cardiology and echocardiography, with >10 years of experience in advanced echocardiographic imaging). All measurements were independently re-evaluated by a second subspecialist (Z.Y.V.-O.); discrepancies were resolved by consensus.

LV morphology was evaluated in standard apical views (4-chamber, 2-chamber, and 3-chamber) following ASE/EACVI recommendations [18]. Images were optimized for a frame rate between 60 and 100 frames per second, as recommended by the EACVI/ASE/Industry Task Force consensus document on speckle-tracking standardization [19]. Care was taken to avoid foreshortening and to ensure full myocardial visualization throughout the cardiac cycle.

Speckle-tracking analysis was performed offline using EchoPAC software (GE Healthcare, version 112.5) on at least three cardiac cycles. The software-generated automated tracking was visually inspected on a segment-by-segment basis, and manual adjustments to the region of interest were performed when necessary to ensure adequate tracking quality. Segments with persistently inadequate tracking despite manual adjustment were excluded from the analysis. LV global longitudinal strain (GLS) was computed as the average peak systolic strain from the three apical views using a 17-segment model.

Non-invasive myocardial work (MW) indices were quantified using the vendor-specific pressure–strain loop module based on the method validated by Russell et al. [13,14]. After computing LV GLS, the timing of aortic and mitral valve events was indicated via echocardiography and brachial systolic blood pressure was entered; the software then generated a non-invasive LV pressure–strain loop for each segment. Peak LV systolic pressure was assumed to equal peak brachial systolic blood pressure. Strain and pressure data were synchronized using the ECG R-wave as a common time reference. MW was evaluated from mitral valve closure to mitral valve opening. From these loops, the following global indices were derived: global work index (GWI, total area of the pressure–strain loop), global constructive work (GCW, work performed during shortening in systole plus work during lengthening in isovolumic relaxation), global wasted work (GWW, work performed during lengthening in systole plus work during shortening in isovolumic relaxation), and global work efficiency (GWE, GCW divided by the sum of GCW and GWW).

To address chamber-level mechanics beyond the LV, left atrial reservoir strain (PALS) and RV free-wall longitudinal strain (RVFW strain) were also analyzed. Strain analysis was successfully performed in all 34 patients to obtain LV GLS measurements. However, not all advanced parameters were analyzable in every patient: MW indices (GWI, GCW, GWW, and GWE) were obtained in 29/34 (85%; PH: 15/16, SH: 14/18), PALS in 31/34 (91%; PH: 16/16, SH: 15/18), and RVFW strain in 30/34 (88%; PH: 16/16, SH: 14/18). Missing data was attributable to suboptimal apical views or insufficient frame rates for reliable tracking in the remaining cases.

Formal inter- and intra-observer variability analyses were not performed in this pilot study, which is acknowledged as a limitation. However, the single-operator acquisition and dual-review protocol minimizes measurement variability. Formal reproducibility assessments are recommended for future larger-scale studies.

### 2.7. Echocardiographic Phenotype Classification

Echocardiographic phenotypes were classified by a single echocardiographer (L.A.V.-Q.) and independently verified by a second subspecialist (Z.Y.V.-O.); discrepancies were resolved by consensus. Four phenotypes were defined according to the following operational criteria, which aligned with ASE/EACVI 2015 guidelines [18]:

Normal phenotype: normal LV cavity dimensions and wall thickness according to sex-specific reference values, LVEF ≥ 52% (biplane Simpson) or ≥55% (3D), grade 0–I diastolic function, and no significant valvular disease.

Dilated phenotype: increased LV end-diastolic volume index (LVEDVi > 75 mL/m^2^ in men, >62 mL/m^2^ in women), with or without reduced LVEF (<52%), consistent with a dilated cardiomyopathy pattern.

Restrictive phenotype: normal or reduced LV cavity size with evidence of grade II–III diastolic dysfunction, defined by E/e′ >14, restrictive transmitral filling pattern (E/A > 2, deceleration time < 150 ms), and/or elevated left atrial volume index (LAVi > 34 mL/m^2^), with preserved or mildly reduced LVEF.

RV/Pulmonary Hypertension (RV/PH) phenotype: RV dilatation (basal RV diameter > 41 mm) and/or RV systolic dysfunction (TAPSE < 17 mm, RV S′ < 9.5 cm/s), with estimated PASP > 35 mmHg, in the absence of a dominant left-sided cardiomyopathy as the primary phenotype.

For prespecified analyses, “advanced phenotype” was defined as any non-normal phenotype (Dilated, Restrictive, or RV/PH).

### 2.8. Statistical Analysis

Continuous variables were tested for normality using the Kolmogorov–Smirnov test and are presented as means ± standard deviations (SDs) when normally distributed or as medians (interquartile ranges (IQRs)) otherwise; categorical variables are reported as counts and percentages. Between-group comparisons (PH vs. SH) were performed using Student’s *t*-test or Mann–Whitney U test for continuous variables and chi-square or Fisher’s exact test for categorical variables, as appropriate.

Associations between iron burden and myocardial function were assessed using Spearman rank correlation coefficients. In the expanded analyses, Spearman correlations were computed between ferritin definitions (at echocardiography, maximum, and most recent) and MW indices (GWI, GCW, GWW, and GWE), and between BNP and MW indices, in the overall cohort and stratified by hemochromatosis type (PH and SH) when feasible. Additional exploratory correlations were performed between ferritin/BNP and deformation indices.

To provide an integrated view of relationships among biomarkers, hemodynamics, remodeling, and myocardial mechanics, a Spearman correlation matrix was generated including ferritin, BNP, PASP, LAVi, GLS, PALS, RVFW strain, and MW indices. All tests were two-tailed, and *p* < 0.05 was considered statistically significant. Analyses were performed using Python (version 3.10.12) with NumPy (version 1.26.4), SciPy (version 1.15.2), and Matplotlib (version 3.10.1).

Given the limited sample size (*n* = 34) and the number of correlation analyses performed across multiple ferritin definitions, MW indices, and subgroups, no correction for multiple comparisons was applied. This approach is consistent with the exploratory, hypothesis-generating design of this pilot study, but it increases the risk of type I errors. All reported *p*-values should therefore be interpreted with caution, and the observed associations require confirmation in adequately powered prospective studies.

## 3. Results

### 3.1. Study Population and Iron Burden

A total of 34 patients were included in the study (mean age: 65 ± 15 years; 50% female), comprising 16 patients with PH and 18 with SH. Baseline demographic, clinical, and biochemical characteristics are summarized in Table 1.

In the PH group, genetic testing identified HFE gene mutations in all cases, with C282Y as the most prevalent variant (27%), followed by H63D (12%). The etiology of the SH group was heterogeneous, with myelodysplastic syndromes (54%) and hemoglobinopathies such as β-thalassemia (28%) being the predominant causes.

There was a marked disparity in systemic iron burden between groups. SH patients showed significantly higher maximum serum ferritin (median 2954 ng/mL vs. 444 ng/mL, *p* = 0.001) and elevated BNP levels (median 93 pg/mL vs. 13.5 pg/mL, *p* = 0.001) compared with PH patients. Management strategies differed by etiology: 81% of PH patients underwent therapeutic phlebotomy, whereas 67% of SH patients received iron chelation therapy (predominantly deferasirox). The median interval from diagnosis to echocardiographic evaluation was 4.5 years (range 0–33), with a notable difference between groups: 10 years (range 0–33) for PH patients vs. 2 years (range 0–15) for SH patients, reflecting the characteristically delayed recognition of hereditary hemochromatosis. During follow-up, six deaths occurred, all in the SH group (33% mortality vs. 0% in PH group, *p* = 0.01).

### 3.2. Echocardiographic Phenotypes

**Figure 1:**
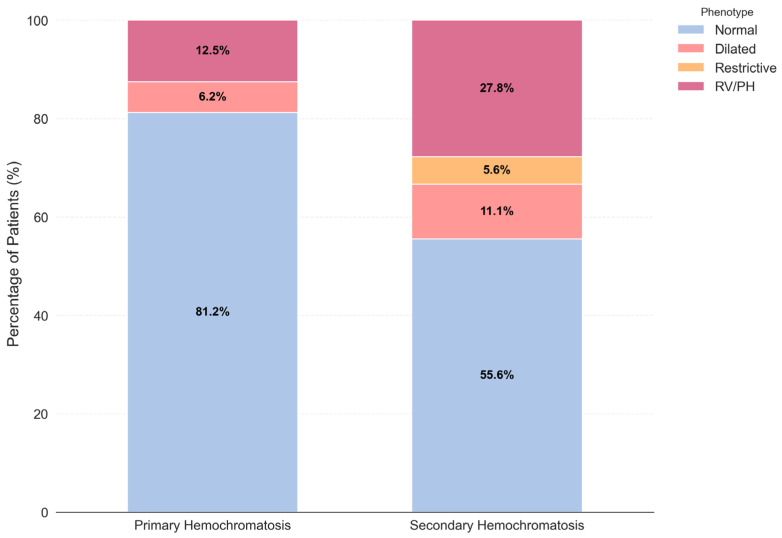
Distribution of echocardiographic phenotypes in PH vs. SH. Stacked bar chart illustrating the proportion of Normal, Dilated, Restrictive, and RV/pulmonary hypertension (RV/PH) phenotypes stratified by etiology. SH group exhibited a significantly higher prevalence of advanced cardiac phenotypes compared with PH group.

The distribution of echocardiographic phenotypes differed markedly by etiology (Figure 1).

PH patients exhibited a predominantly benign profile, with 81% classified as Normal phenotypes. Advanced phenotypes were rare in PH patients: one patient (6%) showed a Dilated phenotype and two (12%) presented RV/PH phenotypes. In contrast, SH patients displayed a significantly more aggressive cardiac phenotype. Only 55% presented a Normal echocardiographic pattern, whereas 45% showed advanced phenotypes: RV/PH (27%), Dilated (11%), and Restrictive (5%).

### 3.3. Conventional Echocardiography and Myocardial Mechanics

Conventional systolic function remained largely preserved in both groups, with similar 3D LVEFs (PH 61.5% (50–72) vs. SH 60.5% (36–70), *p* = 0.82) (Table 2).

However, SH showed trends toward greater structural remodeling, including higher LAVi (33.8 vs. 24.5 mL/m^2^, *p* = 0.06) and elevated PASP (36 vs. 28 mmHg, *p* = 0.07), though these did not reach statistical significance. Diastolic function indices (average e′ and E/e′ ratio) and RV function (TAPSE) were comparable between groups.

Advanced myocardial mechanics are also presented in Table 2. LV GLS did not differ significantly between PH and SH patients (−19.9% vs. −20.0%, *p* = 0.96). Similarly, PALS (28.6% vs. 28.1%, *p* = 0.78) and RVFW strain (−24.0% vs. −24.8%, *p* = 0.68) were comparable between groups. MW indices were also similar between PH and SH patients, including GWI (1797 vs. 1753 mmHg%, *p* = 0.76), GCW (2012 vs. 2092 mmHg%, *p* = 0.58), GWW (85 vs. 82 mmHg%, *p* = 0.91), and GWE (96% vs. 96%, *p* = 0.88).

#### 3.3.1. Ferritin and Global Work Index

**Figure 2:**
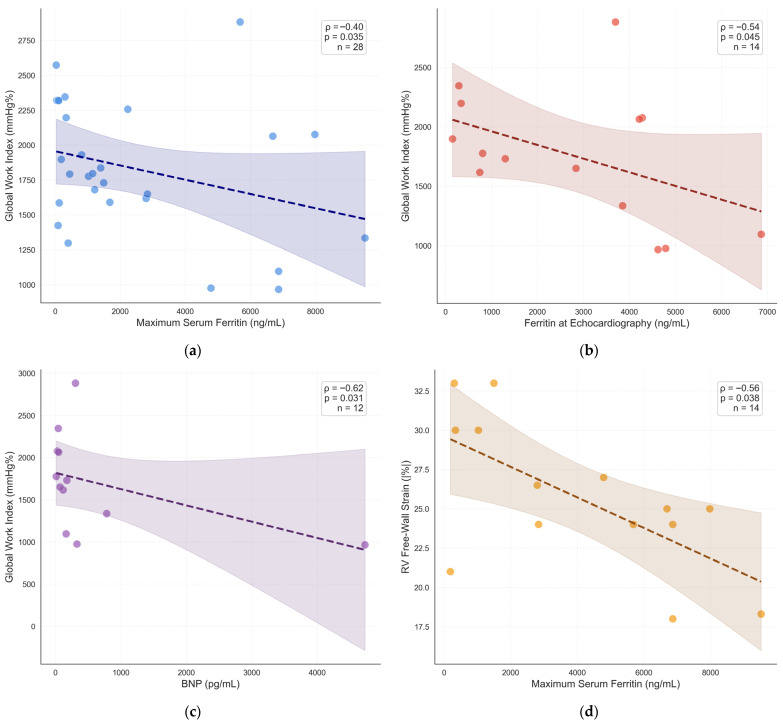
Associations between iron burden, neurohormonal activation, and myocardial mechanics. (**a**) Scatter plot showing the inverse correlation between maximum serum ferritin and GWI in the overall cohort (Spearman ρ = −0.40, *p* = 0.035; *n* = 28). (**b**) Scatter plot demonstrating a stronger inverse association between ferritin at echocardiography and GWI in SH patients (ρ = −0.54, *p* = 0.045; *n* = 14). (**c**) Scatter plot showing the inverse relationship between BNP and GWI in SH patients (ρ = −0.62, *p* = 0.031; *n* = 12). (**d**) Scatter plot illustrating the positive correlation between maximum ferritin and RVFW strain in SH patients (ρ = 0.56, *p* = 0.038; *n* = 14), indicating less favorable RV mechanics with higher iron burden. Each point represents one patient. Solid lines indicate the Spearman regression trend; shaded areas represent 95% confidence bands.

Across the entire cohort, GWI showed significant inverse correlations with ferritin measured at/near the time of echocardiography (ρ = −0.397, *p* = 0.036; *n* = 28) and with maximum ferritin (ρ = −0.399, *p* = 0.035; *n* = 28) (Figure 2a, Table 3), indicating that a higher systemic iron burden is associated with reduced global myocardial work efficiency.

In contrast, most recent ferritin did not correlate significantly with GWI (ρ = −0.150, *p* = 0.446). Subgroup analysis revealed that this relationship was driven predominantly by the SH cohort. In SH patients, ferritin at echocardiography demonstrated a stronger inverse association with GWI (ρ = −0.543, *p* = 0.045; *n* = 14) (Figure 2b), whereas the correlation with maximum ferritin did not reach statistical significance (ρ = −0.398, *p* = 0.158). In PH patients, no significant correlations were observed between ferritin metrics and GWI (all *p* > 0.26) (Table 3).

Correlations between ferritin and GCW followed a similar direction but did not reach statistical significance in any subgroup, though a trend with ferritin at echocardiography was observed in SH patients (ρ = −0.464, *p* = 0.095) (Table 3). No significant associations were identified between ferritin and GWW or GWE.

#### 3.3.2. BNP and Myocardial Work

In the overall cohort, BNP was not significantly associated with MW indices. However, within the SH subgroup, higher BNP levels were significantly associated with lower GWI (ρ = −0.622, *p* = 0.031; *n* = 12) (Figure 2c, Table 3), indicating that neurohormonal activation tracked with reduced global myocardial work in secondary iron overload. A similar trend was observed with GCW (ρ = −0.545, *p* = 0.067). In PH patients, BNP did not correlate with MW indices (Table 3).

### 3.4. Associations Between Iron Burden and Chamber Deformation

Correlations between ferritin/BNP and chamber-specific deformation indices are presented in Table 4.

In the overall cohort, neither ferritin nor BNP correlated significantly with LV GLS, PALS, or RVFW strain. Subgroup analyses revealed distinct patterns. In SH patients, higher maximum ferritin was associated with less favorable (less negative) RVFW strain values (ρ = 0.559, *p* = 0.038; *n* = 14) (Figure 2d), suggesting that iron burden may impair RV mechanics in secondary disease. A trend was also observed between ferritin and LV GLS in SH patients (ρ = 0.427, *p* = 0.077) and between ferritin and PALS (ρ = −0.448, *p* = 0.094).

In PH, PALS showed a positive correlation with maximum ferritin (ρ = 0.632, *p* = 0.011; *n* = 15), which warrants cautious interpretation given the small sample size and counterintuitive directionality (Table 4).

### 3.5. Myocardial Work and Echocardiographic Phenotype Severity

**Figure 3:**
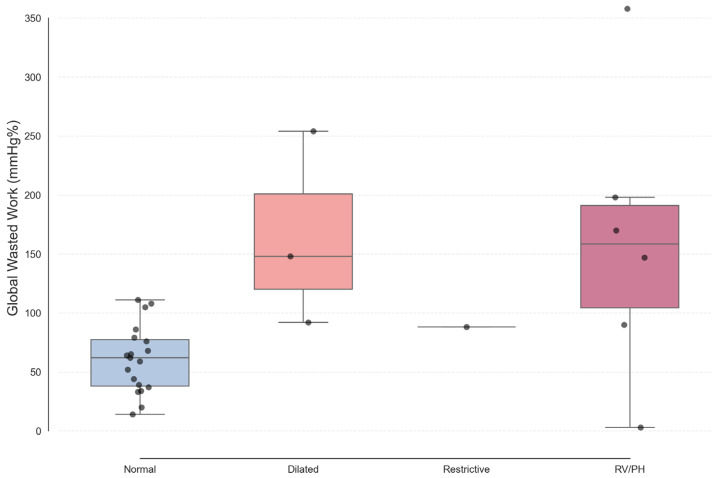
GWW across clinical phenotypes. Box-and-whisker plots demonstrate significantly higher GWW in patients with advanced echocardiographic phenotypes (Dilated and RV/PH) compared with a Normal phenotype, consistent with greater mechanical inefficiency in more advanced structural involvement. Grey dots represent individual data points.

When stratified by echocardiographic phenotype, GWW was markedly higher in patients with advanced phenotypes (Dilated and RV/PH) compared with Normal phenotypes (mean 162 vs. 61 mmHg%), highlighting greater mechanical inefficiency in structurally advanced disease (Figure 3).

### 3.6. Integrated Correlation Structure

**Figure 4:**
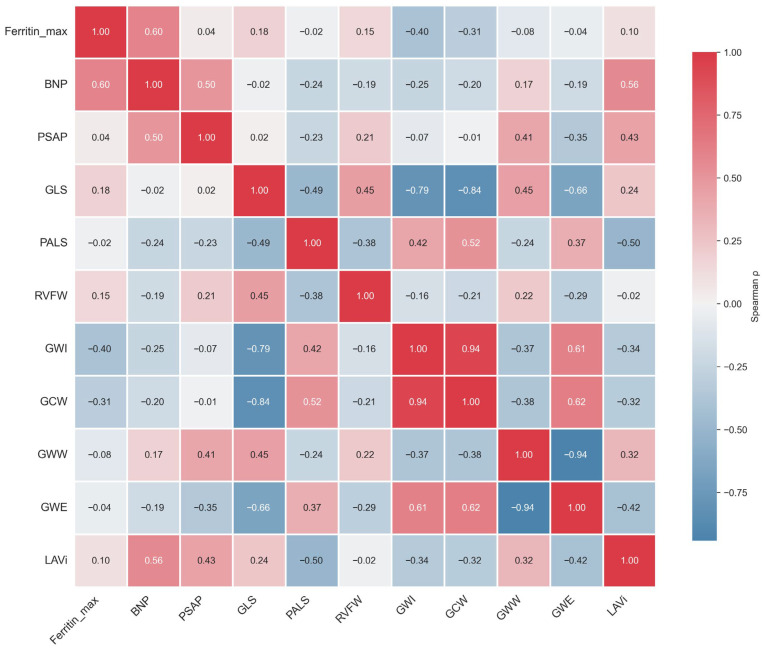
Integrated correlation matrix of iron burden, biomarkers, hemodynamics, chamber remodeling, and myocardial mechanics. Spearman correlation heatmap **including** maximum ferritin, BNP, PASP, LAVi, LV GLS, PALS, RVFW strain, and MW indices (GWI, GCW, GWW, and GWE). Color intensity reflects the magnitude of the Spearman correlation coefficient (ρ). Non-significant correlations are displayed without symbols. Notable associations include ferritin–BNP (ρ = 0.60), BNP–LAVi (ρ = 0.56), GLS–GWI (ρ = −0.79), and GWW–GWE (ρ = −0.94), supporting internal consistency of the mechanics framework.

A Spearman correlation matrix summarizing relationships among biomarkers, hemodynamics, remodeling, and myocardial mechanics is presented in Figure 4.

Maximum ferritin correlated strongly with BNP (ρ = 0.60), supporting concordance between systemic iron burden and neurohormonal activation. BNP correlated with PASP (ρ = 0.50) and LAVi (ρ = 0.56), consistent with hemodynamic consequences of iron-mediated cardiomyopathy. Expected strong interrelationships were observed among myocardial mechanics indices, including GLS–GWI (ρ = −0.79), GLS–GCW (ρ = −0.84), and GWW–GWE (ρ = −0.94), confirming internal consistency of the mechanics framework. GWI showed an inverse correlation with LAVi (ρ = −0.34), further supporting the relationship between reduced myocardial work and atrial remodeling.

### 3.7. Exploratory Survival Analysis

Over a median follow-up of approximately 2 years (range 0–7), six deaths occurred, all within the SH subgroup (33% of SH patients). Patients who died during follow-up exhibited lower mean GWI (1424.6 vs. 1914.9 mmHg%) and less negative mean GLS (−17.6% vs. −20.6%) compared with survivors, though formal prognostic modeling was not performed given the small number of events.

## 4. Discussion

In this retrospective, hypothesis-generating pilot study, we characterized distinct cardiovascular phenotypes in PH and SH and explored whether non-invasive MW indices provide complementary information beyond conventional echocardiographic parameters such as LVEF and GLS. Although global systolic function and strain were largely preserved across groups, increasing iron burden was associated with a progressive reduction in GWI scores, particularly in SH patients, raising the hypothesis that MW may serve as a potential early marker of subclinical myocardial inefficiency [5,14,20].

In line with current guidelines, HFE mutations were the predominant cause of PH in our study population, with C282Y and H63D as the most frequent variants, and most PH patients exhibited a benign cardiovascular profile with normal echocardiograms [1,15,21]. This likely reflects early diagnosis and effective iron removal therapy, as PH patients had substantially lower maximum ferritin levels and were predominantly managed with therapeutic phlebotomy, which is known to prevent or reverse cardiac involvement when instituted before advanced disease [1,15,21]. In contrast, SH, driven mainly by myelodysplastic syndromes and transfusion dependent hemoglobinopathies, was associated with markedly higher iron burden, elevated BNP, and a higher prevalence of non-normal echocardiographic phenotypes, consistent with the more aggressive clinical course de-scribed in transfusional IOC [2,3,6,22].

Historically, restrictive filling patterns and diastolic dysfunction were considered early hallmarks of IOC, particularly in thalassemia major [6,9]. In our series, however, restrictive physiology was rare, whereas RV/pulmonary hypertension was the most common advanced phenotype in SH patients, suggesting that contemporary SH populations are older, with heterogeneous etiologies and variable transfusion burdens, and may follow a remodeling trajectory different from classic thalassemia groups [6,7,9]. The clustering of high ferritin, elevated BNP, increased PASP, and atrial enlargement supports a pathophysiological continuum in which iron-mediated toxicity, neurohormonal activation, and pulmonary vascular disease converge, even when LVEF remains within normal ranges [3,4,5,6,7].

### 4.1. Myocardial Mechanics: Strain and Work

Neither GLS nor RVFW strain differed significantly between the PH and SH groups, and correlations between ferritin and GLS did not reach statistical significance, consistent with prior studies showing that GLS can detect subclinical dysfunction in iron overload but may be heterogeneous across patients and loading conditions [8,12,20,23]. By contrast, GWI showed a modest but statistically significant inverse correlation with ferritin in the overall cohort and a stronger association with ferritin at echocardiography in SH patients, whereas no significant correlations were observed in PH patients, suggesting that MW may be particularly sensitive to the cumulative metabolic impact of transfusional iron [12,14,20,23].

The potential complementary value of MW indices relative to GLS may be explained by the incorporation of afterload into the pressure–strain loop framework, thereby providing a more physiologically complete assessment of myocardial energetics [12,24]. In IOC, oxidative stress and mitochondrial dysfunction impair ATP generation and calcium handling before significant fiber shortening is compromised, phenomena that may be better captured by work inefficiency (elevated GWW) than by strain alone [4,5,6]. The inverse relationship between GWI and LAVi, together with markedly higher GWW in advanced phenotypes, is consistent with the concept that iron-induced oxidative stress and fibrosis translate into inefficient pressure–strain loops before overt systolic failure becomes apparent [4,5,6,14].

It is important to emphasize that the correlation coefficients reported in this study, while statistically significant in specific subgroups, are modest in magnitude (ρ = −0.40 to −0.54) and associated with considerable scatter (Figure 2). These associations reflect group-level trends and should not be interpreted as having predictive value at the individual patient level. The wide dispersion underscores the multifactorial nature of myocardial dysfunction in iron overload, where iron burden represents only one of several determinants. Accordingly, GWI should not be considered a stand-alone surrogate of iron-mediated cardiac injury, but rather one component within a multiparametric echocardiographic assessment. Future studies with larger sample sizes and CMR T2* correlation are needed to define clinically meaningful thresholds.

### 4.2. The Paradox of Similar MW Between PH and SH Despite Ferritin Differences

The apparent paradox that SH patients exhibited substantially higher ferritin levels than PH patients yet similar group-level MW indices reflect the distinct natural histories and clinical management of the two conditions rather than invalidating the utility of GWI [1,2,15,24]. In PH, early genetic diagnosis and prompt initiation of phlebotomy result in effective iron depletion before significant cardiac involvement [15,21,24]. In SH, iron accumulation is chronic and transfusion-dependent, resulting in wide variability in cardiac involvement despite uniformly elevated ferritin [6,22,24]. The inverse correlation between ferritin and GWI emerged predominantly within SH patients, particularly when ferritin was measured contemporaneously with echocardiography, underscoring that MW indices capture intragroup gradients of toxicity rather than simply distinguishing between etiologies [12,14,20].

It must be acknowledged that no statistically significant differences in MW indices were observed. The reported associations are based on within-group correlations of modest magnitude, which limit the strength of inferences regarding the incremental value of MW over conventional parameters. These findings should be interpreted as hypothesis-generating rather than confirmatory.

An important caveat is that serum ferritin is an imperfect surrogate of myocardial iron content. Ferritin levels are elevated by inflammation, hepatic dysfunction, and malignancy independently of true iron stores [16,25], a limitation particularly relevant for SH patients with heterogeneous hematologic conditions. The correlations reported here therefore reflect associations between systemic iron markers and myocardial mechanics, not necessarily between myocardial iron content and cardiac function.

### 4.3. Chamber-Specific Deformation

In SH, higher maximum ferritin was associated with less negative RVFW strain, consistent with early RV involvement in the setting of pulmonary hypertension and pressure overload [3,6,7]. This pattern parallels the predominance of RV/PH phenotypes and supports the vulnerability of the RV to combined pressure and volume overload in secondary iron overload [2,3,6,7].

PALS did not differ significantly between groups and was available in 31 of 34 patients (91%), limiting definitive conclusions regarding atrial mechanics. The positive correlation between PALS and ferritin in the PH group is counterintuitive and likely reflects sampling variability in a small, clinically stable subgroup rather than a true adaptive response [8,21].

### 4.4. Clinical Implications

From a clinical standpoint, the coexistence of preserved LVEF with reduced GWI calls into question the reliance on LVEF alone for surveillance of IOC and supports a multiparametric approach combining strain, MW indices, biomarkers, and, when available, CMR T2* imaging [7,18,25]. In transfusion-dependent SH, MW indices could complement conventional parameters to identify patients who may benefit from intensified monitoring even when LVEF and GLS appear preserved, although this remains to be demonstrated prospectively.

Olivieri et al. established that maintaining serum ferritin below 2500 ng/mL is associated with excellent prognosis in thalassemia major [22]. Our findings raise the hypothesis that MW indices may serve as functional correlations of biochemical iron thresholds, but whether specific GWI or GWW cut-off values carry clinical significance cannot be determined from the present cross-sectional data.

Future prospective studies incorporating serial echocardiographic assessments would be needed to determine whether MW indices can track temporal changes in myocardial function in response to iron-removal therapy. If correlation with CMR T2* is confirmed in larger cohorts, these preliminary findings could suggest a potential role for MW indices in the longitudinal monitoring of iron overload cardiomyopathy, complementing existing surveillance strategies. Integration of standardized CMR T2* mapping to directly quantify myocardial iron alongside serial MW assessment would clarify whether MW tracks cardiac iron deposition or systemic burden, and whether MW-guided strategies improve outcomes compared with conventional monitoring alone [25,26,27]. A prospective study correlating MW indices with CMR T2*-derived myocardial iron concentration represents the natural next step in validating the associations identified in this exploratory analysis.

### 4.5. Limitations

This study has inherent limitations related to its retrospective, single-center design and modest sample size (*n* = 34). No correction for multiple comparisons was applied, consistent with the exploratory design, and some reported associations may represent chance findings. The observed correlations, while statistically significant in selected subgroups, are modest (ρ = −0.40 to −0.54) and should be interpreted as group-level trends rather than individual predictors. Serum ferritin is an acute-phase reactant [16], and residual confounding by subclinical inflammatory states cannot be excluded, particularly in the heterogeneous SH group.

CMR with T2* mapping, the reference standard for myocardial iron quantification [25,26,27], was available in 12 of 34 patients (35%) but was not acquired under a standardized protocol, preventing direct correlation between echocardiographic indices and tissue-level iron content. Not all advanced parameters were analyzable in every patient: MW indices were obtained in 29/34 (85%), PALS in 31/34 (91%), and RVFW strain in 30/34 (88%), owing to suboptimal apical views or insufficient frame rates in the remaining cases.

MW analysis relied on brachial blood pressure as a surrogate for LV systolic pressure using a single vendor-specific platform [14,16,28,29]. Blood pressure was comparable between groups (Table 1), but sensitivity analyses adjusted for antihypertensive therapy were not performed, an inherent constraint of the non-invasive pressure–strain loop methodology [24]. Formal inter- and intra-observer reproducibility was not assessed, though the single-operator acquisition and dual-review protocol mitigates this concern. Follow-up dates were recorded as calendar years, limiting temporal precision. The small number of events (*n* = 6) precluded formal prognostic modeling.

## 5. Conclusions

In this retrospective pilot study, PH and SH were associated with divergent IOC phenotypes despite similarly preserved LVEFs and GLSs at the group level. The PH group, managed mainly with therapeutic phlebotomy, showed lower iron burdens, predominantly normal echocardiograms, and no observed deaths, whereas the SH group presented markedly higher ferritin and BNP, a higher prevalence of advanced phenotypes, particularly RV/PH, and all recorded mortalities.

MW analysis may provide complementary information beyond conventional echocardiography. GWI showed a modest inverse correlation with iron burden markers in selected subgroups, and GWW was higher in advanced phenotypes, raising the hypothesis that pressure–strain loop-derived indices could capture subclinical myocardial inefficiency not reflected by LVEF or GLS alone. However, no significant between-group differences in MW indices were observed, and the reported associations are of modest magnitude with considerable scatter, precluding individual-level prediction. These findings are exploratory, derived from a small single-center cohort without systematic CMR T2* validation, and should be interpreted as hypothesis-generating. Confirmation in larger, prospective, multicenter studies incorporating CMR T2* mapping and serial echocardiographic assessments is warranted before clinical recommendations can be formulated.

## Figures and Tables

**Table 1:** Baseline demographic, clinical, and biochemical characteristics stratified by hemochromatosis type. Data are presented as n (%), mean ± SD, or median (IQR), as appropriate. Abbreviations: PH, primary hemochromatosis; SH, secondary hemochromatosis; BNP, B-type natriuretic peptide.

Variable	PH (*n* = 16)	SH (*n* = 18)	*p*-Value
**Demographics**			
Age, years (Range)	63 (28–78)	63 (23–85)	0.81
Female Sex, n (%)	7 (44%)	10 (56%)	0.48
Disease duration, median years (range)	10 (0–33)	2 (0–15)	—
**Comorbidities**			
Hypertension, n (%)	3 (19%)	5 (28%)	0.54
Diabetes Mellitus, n (%)	3 (19%)	4 (22%)	0.81
Dyslipidemia, n (%)	4 (25%)	1 (6%)	0.12
Hypothyroidism, n (%)	4 (25%)	1 (6%)	0.12
**Biochemical Profile**			
Hemoglobin, g/dL	16 (11–18)	8 (6–14)	<0.001
Max Serum Ferritin, ng/mL	444 (34–3168)	2954 (187–9519)	0.001
Transferrin Saturation, %	52 (21–87)	74 (24–89)	0.08
BNP, pg/mL	13.5 (5–125)	93 (12–4730)	0.001
Troponin I, ng/mL	2.3 (2–6)	7.7 (2–1712)	0.09
**Management & Outcomes**			
Iron Chelation Therapy, n (%)	1 (6%)	12 (67%)	<0.001
Therapeutic Phlebotomy, n (%)	13 (81%)	0 (0%)	<0.001
All-cause Mortality, n (%)	0 (0%)	6 (33%)	0.01

**Table 2:** Conventional and advanced echocardiographic parameters in PH vs. SH. Data are presented as median (range) or median (IQR), as appropriate. Abbreviations: LVEF, left ventricular ejection fraction; LAVi, left atrial volume index; PASP, pulmonary artery systolic pressure; TAPSE, tricuspid annular plane systolic excursion; GLS, global longitudinal strain; PALS, peak atrial longitudinal strain; RVFW, right ventricular free-wall strain; GWI, global work index; GCW, global constructive work; GWW, global wasted work; GWE, global work efficiency; PH, primary hemochromatosis; SH, secondary hemochromatosis. Not all advanced deformation parameters were analyzable in every patient owing to suboptimal apical views or insufficient frame rates for reliable speckle tracking. The number of patients with available measurements (n) is indicated for each parameter.

Variable	n (PH/SH)	PH (*n* = 16)	SH (*n* = 18)	*p*-Value
**Conventional Parameters**				
LVEF (3D), %	16/18	61.5 (50–72)	60.5 (36–70)	0.82
LV End-Diastolic Volume, mL	16/18	76 (41–115)	92.9 (51–132)	0.14
LAVi, mL/m^2^	16/18	24.5 (16–44)	33.8 (35–59)	0.06
Mitral Average e′, cm/s	16/18	9.2 (5–13)	8.7 (5–15)	0.45
Average E/e′ Ratio	16/18	7.9 (4–12)	8.5 (4–18)	0.65
TAPSE, mm	16/18	21 (17–28)	23 (17–29)	0.28
PASP, mmHg	16/18	28 (20–35)	36 (22–65)	0.07
**Advanced Mechanics**				
LV GLS, %	16/18	−19.9 (−27 to −15)	−20.0 (−25 to −14)	0.96
PALS, %	16/15	28.6 (20–49)	28.1 (10–47)	0.78
RVFW Strain, %	16/14	−24.0 (−30 to −16)	−24.8 (−33 to −18)	0.68
GWI, mmHg%	15/14	1797 (1298–2574)	1753 (966–2883)	0.76
GCW, mmHg%	15/14	2012 (1486–2834)	2092 (1137–3348)	0.58
GWW, mmHg%	15/14	85 (14–358)	82 (25–198)	0.91
GWE, %	15/14	96 (84–99)	96 (86–99)	0.88

**Table 3:** Spearman correlations between MW indices and iron burden/neurohormonal markers stratified by cohort and hemochromatosis type. Values are reported as Spearman ρ coefficient, *p*-value, and number of paired observations (n). Abbreviations: Ferritin_dx, ferritin at/near echocardiography; Ferritin_max, maximum recorded ferritin; Ferritin_rec, most recent ferritin; BNP, B-type natriuretic peptide; GWI, global work index; GCW, global constructive work; GWW, global wasted work; GWE, global work efficiency; All, total cohort; PH, primary hemochromatosis; SH, secondary hemochromatosis. Ferritin at echocardiography: serum ferritin obtained within ±30 days of the echocardiographic study; when multiple values were available, the closest measurement was used.

Group	MW Index	Marker	*n*	Spearman ρ	*p*-Value
All	GWI	Ferritin_dx	28	−0.397	0.036
		Ferritin_max	28	−0.399	0.035
		Ferritin_rec	28	−0.150	0.446
		BNP	23	−0.250	0.251
	GCW	Ferritin_dx	28	−0.282	0.146
		Ferritin_max	28	−0.309	0.109
		Ferritin_rec	28	−0.076	0.7
		BNP	23	−0.196	0.371
	GWW	Ferritin_dx	28	−0.016	0.934
		Ferritin_max	28	−0.082	0.678
		Ferritin_rec	28	−0.070	0.725
		BNP	23	0.175	0.426
	GWE	Ferritin_dx	28	−0.071	0.721
		Ferritin_max	28	−0.044	0.824
		Ferritin_rec	28	0.04	0.839
		BNP	23	−0.194	0.375
PH	GWI	Ferritin_dx	14	−0.248	0.392
		Ferritin_max	14	−0.305	0.288
		Ferritin_rec	14	0.323	0.26
		BNP	11	0.352	0.289
	GCW	Ferritin_dx	14	−0.121	0.681
		Ferritin_max	14	−0.182	0.533
		Ferritin_rec	14	0.319	0.267
		BNP	11	0.315	0.345
	GWW	Ferritin_dx	14	−0.112	0.703
		Ferritin_max	14	−0.077	0.794
		Ferritin_rec	14	−0.257	0.375
		BNP	11	−0.146	0.668
	GWE	Ferritin_dx	14	0.023	0.939
		Ferritin_max	14	−0.084	0.776
		Ferritin_rec	14	0.289	0.316
		BNP	11	0.212	0.532
SH	GWI	Ferritin_dx	14	−0.543	0.045
		Ferritin_max	14	−0.398	0.158
		Ferritin_rec	14	−0.451	0.106
		BNP	12	−0.622	0.031
	GCW	Ferritin_dx	14	−0.464	0.095
		Ferritin_max	14	−0.323	0.259
		Ferritin_rec	14	−0.398	0.158
		BNP	12	−0.545	0.067
	GWW	Ferritin_dx	14	0.09	0.759
		Ferritin_max	14	−0.211	0.469
		Ferritin_rec	14	−0.079	0.788
		BNP	12	0.343	0.276
	GWE	Ferritin_dx	14	−0.221	0.447
		Ferritin_max	14	0.011	0.97
		Ferritin_rec	14	−0.086	0.769
		BNP	12	−0.473	0.121

**Table 4:** Spearman correlations between chamber-specific deformation indices and iron burden/BNP stratified by hemochromatosis type and group. Values are reported as Spearman ρ coefficient, *p*-value, and number of paired observations (n). Abbreviations: GLS, left ventricular global longitudinal strain; PALS, peak atrial longitudinal strain; RVFW, right ventricular free-wall strain; Ferritin_max, maximum recorded ferritin; BNP, B-type natriuretic peptide; All, total cohort; PH, primary hemochromatosis; SH, secondary hemochromatosis. Note: The positive correlation between PALS and ferritin in PH patients (ρ = 0.632, *p* = 0.011) should be interpreted cautiously given the small sample size and counterintuitive directionality.

Group	Deformation Index	Marker	*n*	Spearman ρ	*p*-Value
All	GLS	Ferritin_max	33	0.176	0.326
		BNP	27	−0.023	0.909
	PALS	Ferritin_max	30	−0.023	0.902
		BNP	25	−0.243	0.241
	RVFW	Ferritin_max	29	0.147	0.447
		BNP	24	−0.186	0.385
PH	GLS	Ferritin_max	15	0.061	0.829
		BNP	12	−0.519	0.084
	PALS	Ferritin_max	15	0.632	0.011
		BNP	12	0.058	0.858
	RVFW	Ferritin_max	15	0.269	0.332
		BNP	12	−0.466	0.127
SH	GLS	Ferritin_max	18	0.427	0.077
		BNP	15	0.45	0.092
	PALS	Ferritin_max	15	−0.448	0.094
		BNP	13	−0.517	0.07
	RVFW	Ferritin_max	14	0.559	0.038
		BNP	12	0.428	0.166

## Data Availability

The datasets generated and analyzed during the current study are not publicly available due to privacy and ethical restrictions, as they contain sensitive clinical information derived from patient medical records. However, the data are available from the corresponding author upon reasonable request.

## Abbreviations

The following abbreviations are used in this manuscript:

IOC	Iron overload cardiomyopathy
PH	Primary hemochromatosis
SH	Secondary hemochromatosis
LV	Left ventricle
RV	Right ventricle
LVEF	Left ventricular ejection fraction
GLS	Global longitudinal strain
RVFW	Right ventricular free-wall
PALS	Peak atrial longitudinal strain
LAVi	Left atrial volume index
PASP	Pulmonary artery systolic pressure
MW	Myocardial work
GWI	Global work index
GCW	Global constructive work
GWW	Global wasted work
GWE	Global work efficiency
BNP	B-type natriuretic peptide
CMR	Cardiac magnetic resonance
RVPH	Right ventricular/pulmonary hypertension phenotype
SD	Standard deviation
IQR	Interquartile range
ASE	American Society of Echocardiography
EACVI	European Association of Cardiovascular Imaging

## References

[1] Kowdley K.V., Brown K.E., Ahn J., Sundaram V. (2019). ACG Clinical Guideline: Hereditary Hemochromatosis. Am. J. Gastroenterol..

[2] Aronow W.S. (2018). Management of cardiac hemochromatosis. Arch. Med. Sci..

[3] Díez-López C., Comín-Colet J., González-Costello J. (2018). Iron overload cardiomyopathy: From diagnosis to management. Curr. Opin. Cardiol..

[4] Oudit G.Y., Sun H., Trivieri M.G., Koch S.E., Dawood F., Ackerley C., Yazdanpanah M., Wilson G.J., Schwartz A., Liu P.P. (2003). L-type Ca^2+^ channels provide a major pathway for iron entry into cardiomyocytes in iron-overload cardiomyopathy. Nat. Med..

[5] Zhang H., Zhabyeyev P., Wang S., Oudit G.Y. (2019). Role of iron metabolism in heart failure: From iron deficiency to iron overload. Biochim. Biophys. Acta Mol. Basis Dis..

[6] Kremastinos D.T., Farmakis D. (2011). Iron overload cardiomyopathy in clinical practice. Circulation.

[7] Murphy C.J., Oudit G.Y. (2010). Iron-overload cardiomyopathy: Pathophysiology, diagnosis, and treatment. J. Card. Fail..

[8] Palka P., Macdonald G., Lange A., Burstow D.J. (2002). The role of Doppler left ventricular filling indexes and Doppler tissue echocardiography in the assessment of cardiac involvement in hereditary hemochromatosis. J. Am. Soc. Echocardiogr..

[9] Spirito P., Lupi G., Melevendi C., Vecchio C. (1990). Restrictive diastolic abnormalities identified by Doppler echocardiography in patients with thalassemia major. Circulation.

[10] Rozwadowski J.L., Chattranukulchai P., Jellis C.L., Rodriguez L.L., Thomas J.D., Bolen M.A. (2018). Echocardiographic strain imaging in the monitoring of iron overload cardiomyopathy. Echocardiography.

[11] Swiatczak A., Magielski P., Wojtkowska A., Śledzińska M., Marchel M., Jastrzębska-Kurkowska M., Górska-Walecka A. (2023). Subclinical Systolic Dysfunction and Global Longitudinal Strain in Iron Overload. Front. Cardiovasc. Med..

[12] Chen M.R., Chu A.K., Juan Y.H., Wu J.Y., Hung C.C., Wang J.H. (2015). Relation of myocardial systolic mechanics to serum ferritin level as a prognosticator in thalassemia patients undergoing repeated transfusion. Echocardiography.

[13] Russell K., Eriksen M., Aaberge L., Gjesdal O., Hamre H., Vik-Mo H., Edvardsen T., Smiseth O.A. (2012). Non-invasive myocardial work indices in clinical practice. JACC Cardiovasc. Imaging.

[14] Russell K., Eriksen M., Aaberge L., Gjesdal O., Hamre H., Vik-Mo H., Edvardsen T., Smiseth O.A. (2012). A novel clinical method for quantification of regional left ventricular pressure-strain loop area: A non-invasive index of myocardial work. Eur. Heart J..

[15] European Association for the Study of the Liver (EASL) (2022). EASL Clinical Practice Guidelines on haemochromatosis. J. Hepatol..

[16] Cullis J.O. (2011). Diagnosis and management of anaemia of chronic disease: Current status. Br. J. Haematol..

[17] Whelton P.K., Carey R.M., Aronow W.S., Casey D.E., Collins K.J., Dennison Himmelfarb C., DePalma S.M., Gidding S., Jamerson K.A., Jones D.W. (2018). 2017 ACC/AHA/AAPA/ABC/ACPM/AGS/APhA/ASH/ASPC/NMA/PCNA Guideline for the Prevention, Detection, Evaluation, and Management of High Blood Pressure in Adults. Hypertension.

[18] Lang R.M., Badano L.P., Mor-Avi V., Afilalo J., Armstrong A., Ernande L., Flachskampf F.A., Foster E., Goldstein S.A., Kuznetsova T. (2015). Recommendations for cardiac chamber quantification by echocardiography in adults: An update from the American Society of Echocardiography and the European Association of Cardiovascular Imaging. J. Am. Soc. Echocardiogr..

[19] Voigt J.U., Pedrizzetti G., Lysyansky P., Marwick T.H., Houle H., Baumann R., Pedri S., Ito Y., Abe Y., Metz S. (2015). Definitions for a Common Standard for 2D Speckle Tracking Echocardiography: Consensus Document of the EACVI/ASE/Industry Task Force to Standardize Deformation Imaging. Eur. Heart J. Cardiovasc. Imaging.

[20] Abtahi F., Sahlén A., Winter R., Gustafsson B. (2019). Global longitudinal strain as an Indicator of cardiac Iron overload in thalassemia patients. Cardiovasc. Ultrasound.

[21] Candell-Riera J., Lu L., Serés L., Castell-Conesa J., Garcia-del-Castillo H., Soler-Soler J. (1983). Cardiac hemochromatosis: Beneficial effects of iron removal therapy. An echocardiographic study. Am. J. Cardiol..

[22] Olivieri N.F., Nathan D.G., MacMillan J.H., Wayne A.S., Liu P.P., McGee A., Martin M., Koren G., McClelland R.A. (1994). Survival in medically treated patients with homozygous beta-thalassemia. N. Engl. J. Med..

[23] Montanaro C., Sleiman J., Elitok A., Di Stefano S., Musumeci F., Walker J.M. (2017). Myocardial deformation in iron overload cardiomyopathy: Speckle tracking imaging in a beta-thalassemia major population. Intern. Emerg. Med..

[24] Li X., Zhang J., Gu J., Wang J., Shao Y., Wang H., He Y. (2022). Impact of blood pressure changes on myocardial work indices in hypertensive patients in a day. J. Clin. Hypertens..

[25] Piperno A. (2020). Inherited iron overload disorders. Transl. Gastroenterol. Hepatol..

[26] Trimarchi G., Carerj S., Di Bella G., Manganaro R., Pizzino F., Restelli D., Pelaggi G., Lofrumento F., Licordari R., Taverna G. (2024). Clinical Applications of Myocardial Work in Echocardiography: A Comprehensive Review. J. Cardiovasc. Echogr..

[27] Carpenter J.P., He T., Kirk P., Roughton M., Anderson L.J., de Noronha S.V., Sheppard M.N., Porter J.B., Walker J.M., Cappellini M.D. (2011). On T2* magnetic resonance and cardiac iron. Circulation.

[28] Wood J.C. (2007). Magnetic resonance imaging measurement of iron overload. Curr. Opin. Hematol..

[29] Chan J., Edwards N.F.A., Khandheria B.K., Shiino K., Sabapathy S., Anderson B., Chamberlain R., Scalia G.M. (2019). A new approach to assess myocardial work by non-invasive left ventricular pressure-strain relations in hypertension and dilated cardiomyopathy. Eur. Heart J. Cardiovasc. Imaging.

